# Elastic properties and secondary structure formation of single-stranded DNA at monovalent and divalent salt conditions

**DOI:** 10.1093/nar/gkt1089

**Published:** 2013-11-12

**Authors:** Alessandro Bosco, Joan Camunas-Soler, Felix Ritort

**Affiliations:** ^1^SISSA - Scuola Internazionale Superiore di Studi Avanzati, via Bonomea 265, 34136 Trieste, Italy, ^2^Elettra-Sincrotrone Trieste S.C.p.A., Strada Statale 14 - km 163,5 in AREA Science Park, 34149 Basovizza Trieste, Italy, ^3^Departament de Física Fonamental, Universitat de Barcelona, Diagonal 647, 08028 Barcelona, Spain and ^4^CIBER de Bioingeniería, Biomateriales y Nanomedicina, Instituto de Salud Carlos III, Madrid, Spain

## Abstract

Single-stranded DNA (ssDNA) plays a major role in several biological processes. It is therefore of fundamental interest to understand how the elastic response and the formation of secondary structures are modulated by the interplay between base pairing and electrostatic interactions. Here we measure force-extension curves (FECs) of ssDNA molecules in optical tweezers set up over two orders of magnitude of monovalent and divalent salt conditions, and obtain its elastic parameters by fitting the FECs to semiflexible models of polymers. For both monovalent and divalent salts, we find that the electrostatic contribution to the persistence length is proportional to the Debye screening length, varying as the inverse of the square root of cation concentration. The intrinsic persistence length is equal to 0.7 nm for both types of salts, and the effectivity of divalent cations in screening electrostatic interactions appears to be 100-fold as compared with monovalent salt, in line with what has been recently reported for single-stranded RNA. Finally, we propose an analysis of the FECs using a model that accounts for the effective thickness of the filament at low salt condition and a simple phenomenological description that quantifies the formation of non-specific secondary structure at low forces.

## INTRODUCTION

Understanding the elastic behavior of single-stranded DNA (ssDNA) is of great relevance because of its major role in many biological processes, such as replication, recombination, repair, transcription and transposition of DNA (1). In particular, characterizing the elastic response and the formation of secondary structure is an important task in comprehending the physicochemical properties of ssDNA.

ssDNA forms secondary structure by Watson–Crick hydrogen bonding and other non-canonical base pairings (2). The formation of secondary structure and its elastic properties are also highly determined by base-stacking interactions (3–5). Experiments on homopolymeric nucleic acids have been carried out to elucidate these effects (6,7). The experimental techniques generally used for the analysis of secondary structures rely on the differential chemical or enzymatic reactivity of single- and double-stranded regions of nucleic acids, but the structural information obtained from these experiments is averaged over an ensemble of possible structures adopted by the molecule (8,9).

Single molecule experiments (10) make possible to study molecular reactions by observing or measuring the mechanical response of one molecule at a time. By applying mechanical force to the ends of an ssDNA molecule tethered between two surfaces, it is possible to extract accurate information about its elastic properties and the formation of secondary structure. Despite the large number of articles in the single molecule field, the existing literature addressing the mechanical properties of ssDNA is scarce, especially when compared with that focused on double-stranded DNA (dsDNA) (11). Probably this is due to the fact that ssDNA is difficult to manipulate because it interacts with itself by forming hydrogen bonds (4) and binds specifically to surfaces due to the hydrophobicity of the exposed bases (12–15).

Optical tweezers’ experiments with ssDNA were first performed by force-melting a tethered dsDNA molecule in the presence of distilled water or formaldehyde (16) [see (17) for a recent overview of this method]. These experiments showed that the force-extension curve (FEC) of ssDNA strongly depends on the ionic strength, with large deviations from an ideal chain model at high salt concentration. It is generally accepted that this is due to the formation and stabilization of secondary structure in high salt conditions (18). In fact, the experimental FEC at high divalent salt concentrations can be well described using theoretical models of generic semiflexible polymers that are capable of forming hairpins (19). To investigate electrostatic interactions of ssDNA, an approach that avoids the formation of secondary structure was implemented (18,20–22). This was achieved by using a chemical compound that inhibits the formation of hydrogen bonds (glyoxal). By screening base pairing interactions, glyoxal makes possible to study ssDNA as if it were a ‘pure’ polyelectrolyte.

Here we use the blocking oligo method introduced in (23) to mechanically pull ssDNA tethers. In this method, ssDNA is obtained by mechanically unzipping a long DNA hairpin (6770 bp). Once the hairpin is fully unzipped and the ssDNA stretched, an oligonucleotide designed to bind to the loop region is flushed in the fluidics chamber. The binding of the oligo to the tethered ssDNA prevents the re-zipping of the hairpin as the tension is released. In fact, the entropic effect to close 30 bases within a loop and the large persistence length of the dsDNA segment in the loop region creates a large kinetic barrier that must be overcome, thereby inhibiting the refolding of the native structure. This methodology allows us to study the stretching response of ssDNA over a wide range of forces and salt conditions and should be useful to study the interaction of ssDNA with proteins, peptides and other chemical compounds (24).

Here we extend previous mechanical studies of the elastic properties of ssDNA under varying monovalent salt conditions (25) and report experiments at different divalent salt conditions. The elastic properties of ssDNA in presence of divalent ions have been briefly reported in previous works (16,18,26). To the best of our knowledge, this is the first work in which a systematic study based on force spectroscopy methods of the elastic properties of ssDNA at different divalent salt conditions is presented. Studies have been previously reported with both monovalent and divalent salts in the presence of glyoxal (18,20–22).

We analyze the FECs by using the most commonly adopted models describing the elastic properties of semiflexible charged polymers, such as the Worm-like Chain (WLC) (27,28) model and the Extensible Freely-Jointed Chain (Ex-FJC) model (16,29). These models are widely used to describe the elasticity of ssDNA molecules, but they fail to describe the FEC of ssDNA in some conditions (e.g. at low forces for low ionic strength, and at low and intermediate forces for high ionic strength). To circumvent this problem, an approach based on a polynomial expansion fit of the FEC has been previously proposed (30). Here we also test a description of the FECs based on the Thick Chain (TC) model for low salt concentrations and low forces (31,32). This model takes into account the effects of excluded volume and the electrostatic self-repulsion between the phosphate groups along the ssDNA backbone. Finally, we address the behavior of the ssDNA at high monovalent and divalent salt conditions with a simple phenomenological model accounting for the formation of non-specific secondary structure.

Our work contributes to a better understanding of the elastic parameters describing the FECs of ssDNA at different monovalent and divalent salt conditions using analytically tractable formulas. This knowledge should be most useful to extract the base pairing free energies of dsDNA in the presence of divalent ions from mechanical unzipping experiments. This information has already been shown to be essential to characterize the thermodynamics of hybridization of DNA in monovalent salt from unzipping experiments (25).

## MATERIALS AND METHODS

### Molecular construct, experimental set up and salt conditions

All the experiments were performed using a custom-built dual-beam force measuring optical trap as described in previous works (25,33). Experiments with monovalent ions were performed at room temperature (25°C) in a buffer containing TE, pH 7.5 (Tris-HCl 10 mM, ethylenediaminetetraacetic acid 1 mM), 0.01% NaN_3_ and a varying concentration of NaCl (10, 25, 50, 100, 250, 500 and 1000 mM). Experiments with divalent ions were performed at 25°C in a buffer containing 10 mM Tris-HCl, pH 7.5, 0.01% NaN_3_ and a varying concentration of MgCl_2_ (0.5, 1, 2, 4, 10 mM). An additional amount of 9 mM of monovalent cations contributed by the buffer must be added to all NaCl concentrations and considered as potentially competing with divalent cations in the MgCl_2_ case. However, as discussed later, the latter effect turns out to be negligible in the range of divalent salt concentrations studied in this article. The molecular construct used in the experiments consists of a long DNA hairpin (extracted from λ-DNA) of 6838 bp in the stem ending in a tetraloop. The hairpin is inserted between two flanking 29-bp dsDNA handles at both sides that provide the necessary space to carry out the manipulation of the hairpin [25]. By using such short molecular handles, the signal-to-noise ratio is increased (34). λ-DNA (New England Biolabs) is digested with BamHI (New England Biolabs) and phosphorylated at its 5'-ends. The fragment of the dsDNA between positions 41733 and 48502 (the cosR end) is used as the stem of the DNA hairpin [see (24) for details]. Several oligonucleotides (sequence reported in Supplementary Table S1) are used as building blocks for the synthesis of the loop and the handles of the construct. The protocol for assembling the loop and handles with the stem is described in the Supplementary Section S1).

**Figure 1.**
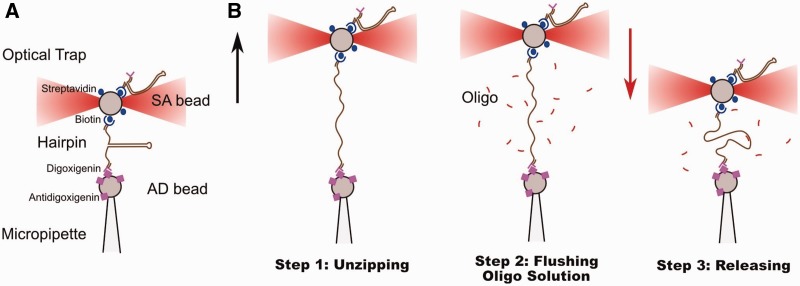
(**A**) Scheme illustrating the connections between a hairpin and the polystyrene beads. SA bead stands for streptavidin-coated bead, whereas AD bead stands for antidigoxigenin-coated bead. Beads and molecules are not in scale. (**B**) Steps of the protocol used in our experiments to obtain FECs of ssDNA.

**Figure 2.**
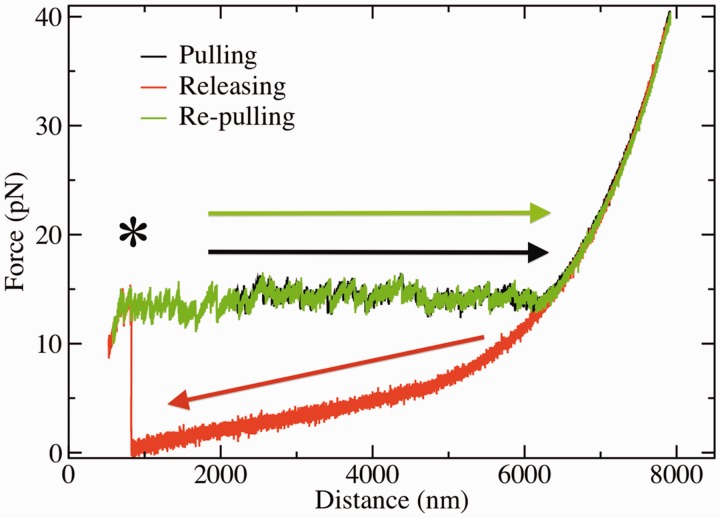
Cycle of pulling, showing the stretching (black) and releasing (red/light gray) parts of the cycle in a hairpin in TE with 100 mM of NaCl. The refolding of the hairpin is observed at low forces (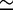
1 pN) as a sudden jump-up in the force (highlighted with an asterisk). The re-pulling curve in the absence of blocking oligo is drawn in green/dark gray [black and green/dark gray curves nearly superimpose due to the quasi-reversibility of the unfolding-folding process, see [(25)].

The two flanking handles are functionalized with biotin on one side and digoxigenin on the other side. Streptavidin-coated polystyrene microspheres (SA beads, 3.0–3.9 μm; G. Kisker GbR, Products for Biotechnology) and antidigoxigenin (Roche Applied Science) antibody-coated bead (AD bead, synthesized from protein G-coated microspheres of 3.2 μm; Spherotech, Libertyville, IL, USA) are used for specific attachments to the DNA molecular construct described earlier. Molecules are tethered to beads via specific attachments (biotin–streptavidin and antidigoxigenin–digoxigenin) and stretched using optical tweezers. SA beads are incubated for 20–30 min with the synthesized hairpins in TE buffer solution at room temperature. An AD bead is positioned and immobilized at the tip of the micropipette by air suction. Then an SA-coated bead is captured in the optical trap and approached to the AD bead in the pipette until a molecular connection is established. Typically, after a few attempts, a stable attachment (resisting up to a force of 40 pN) is obtained. Figure 1A reports a schematic representation of the connections between the beads and the molecular construct. Next, a solution containing a 30-bases oligonucleotide (blocking loop) complementary to the loop region of the hairpin (Supplementary Section S1) is flowed into the chamber. The oligonucleotide solution is previously incubated at 45°C for 15 min to remove any competing secondary structure within the oligonucleotide. We used an oligo concentration ranging from 50 to 250 nM depending on the salt concentration. For each ionic condition, we increased the concentration of the oligo from 50 nM up to a value for which it was possible to obtain the ssDNA releasing pattern in the force signal (Figure 2, red curve). A higher oligo concentration was often needed at low salt concentrations. The highest oligo concentration used was 250 nM for the experiments in TE 10 mM NaCl.

### Unzipping the DNA hairpin

The procedure to measure force–distance curves (FDCs) of ssDNA consists of the following steps. First, a molecule is tethered between the bead captured in the optical trap and the bead immobilized in the tip of the pipette. Then the optically trapped SA bead is moved away from the AD bead at a constant pulling speed of ∼30 nm/s. Meanwhile, the force and relative trap-pipette distance are collected with an acquisition frequency of 200 Hz. The tethered hairpin is unzipped (Figure 1B, step 1) showing a characteristic sawtooth pattern in the FDC with gentle slopes and force rips (Figure 2, green curve). The sawtooth pattern is closely related to the sequence of the hairpin (25). The slopes are related to the elastic response of the ssDNA generated during the unzipping process. When a group of base pairs is released, the tension is relaxed and the force drops. For the sequence used in our experiments, the mean unzipping force lies between 10 and 20 pN varying with salt concentration (Supplementary Information, Figure 2). When the hairpin is completely unzipped, ssDNA is obtained and the force increases monotonically with the distance between the beads (stretching of ssDNA, Figure 2, red curve).

### Transient flushing of the oligonucleotide and releasing of the molecule

Once a force of ∼40 pN is reached (Figure 1B, step 2), a solution of the same buffer containing the blocking loop oligonucleotide is flushed gently through the central channel of the fluidic chamber. The two different buffers can be exchanged by using a valve that regulates the buffer entering the chamber. The beads are then steadily approached at a constant speed. When the oligo binds to the loop region, the refolding of the hairpin is inhibited due to the high kinetic barrier that must be overcome to refold the hairpin structure. We note that the 30 bases oligonucleotide can only specifically bind to the loop region. Therefore, it should not affect the secondary structure formation along the ssDNA, as it is not complementary to any other region of the hairpin. In this case, the releasing of the molecule shows a behavior compatible with a molecule of ssDNA with a number of bases twice the number of base pairs of the hairpin (Figure 1B, step 3). It is often possible to reach forces down to 0.5–1 pN without observing the refolding of the hairpin. Yet, the hairpin sometimes rezips before reaching such low forces, and a sudden jump up to the mean unzipping force of the hairpin is observed (Figure 2, red curve at a distance ∼800 nm, highlighted with an asterisk). By pulling the hairpin again, the sawtooth pattern is recovered. Releasing FDCs have been collected for at least three molecules at each salt condition.

### From FDCs to FECs

Conversion from distances to ssDNA molecular extension is needed to study the ssDNA elastic behavior. FDCs are converted to FECs by subtracting the elastic contribution of the optical trap. In the explored range of forces, the potential energy of the bead in the trap can be approximated by a harmonic well, so the extension or end-to-end distance *x* of the ssDNA molecule can be written as
(1)
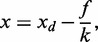

where *x_d_* is the measured distance, *f* is the force and *k* is the stiffness of the optical trap. The stiffness of the optical trap has been estimated for several beads at different salt conditions finding a value of 0.07 ± 0.005 pN/nm (Supplementary Section S2). Then curves have been aligned using the sequence-dependent unzipping pattern of the hairpin previously characterized in (25) and using force data up to 40 pN to fit the elastic response (Supplementary Section S3). Finally, the FECs of ssDNA are aligned with the elastic response of the ssDNA at the rightmost end of unzipping curves (after the sawtooth pattern).

## RESULTS AND DISCUSSION

### Elastic properties of ssDNA

#### Results with monovalent salt (NaCl)

**Figure 3.**
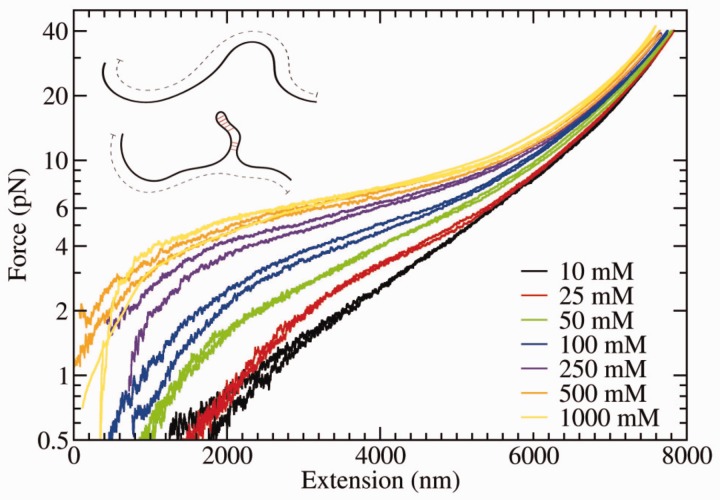
FEC of 13-kb ssDNA at varying NaCl concentration taken at constant pulling speed of ∼30 nm/s and filtered at 2-Hz bandwidth. Inset shows how the formation of secondary structure reduces the apparent contour length of the molecule (dashed lines).

**Figure 4.**
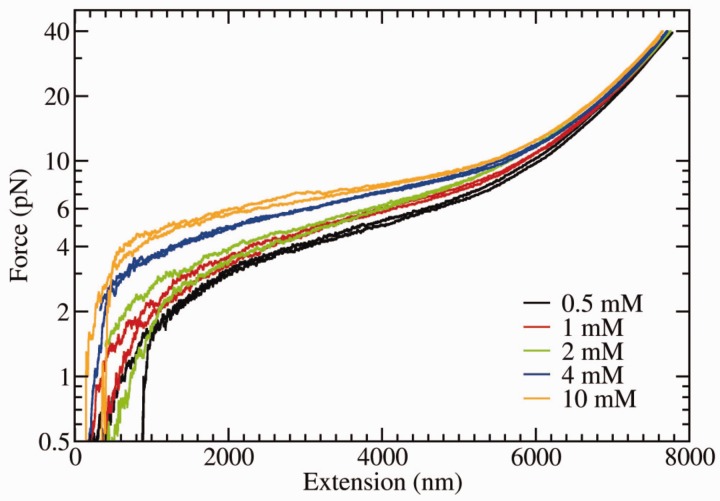
FEC of 13-kb ssDNA at varying MgCl_2_ concentration taken at constant pulling speed of ∼30 nm/s and filtered at 2-Hz bandwidth.

Figure 3 shows the FECs measured at different monovalent salt concentrations filtered at 2-Hz bandwidth. To illustrate the variability in our measurements, we show two different FECs at each salt condition (differences can arise for instance from variability in the geometry of the attachment of each molecule to the bead surface). The force monotonically increases with the extension. At low forces (<10 pN) and high salt (>100 mM), a plateau compatible with the formation of secondary structure is observed (18,19). At low forces (<5 pN), the higher the salt concentration, the shorter the molecular extension. This behavior can be attributed to the formation of secondary structure that reduces the effective length of the ssDNA (Figure 3, inset). The increase in the height of the plateau at higher salt concentration shows that base pairing is stabilized with salt [35]. Moreover, the Debye screening length becomes shorter with increased salt concentration (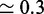 nm at 1 M NaCl), and electrostatic repulsion between the phosphate groups is too short-ranged to prevent base pair formation. The experimental FECs in Figures 3 and 4 do not show the characteristic features observed for single-stranded homopolymeric nucleic acids (6,7) that form helices stabilized by the stacking of consecutive bases. This is not surprising if we consider that our construct contains a random and equally assorted amount of different nucleotides.

#### Results with divalent salt (MgCl_2_)

In Figure 4, we show the FECs at different divalent salt concentrations. For every concentration, two FECs are reported. For monovalent salt, the plateau observed at low forces for high salt concentrations is due to the formation of secondary structure. At equal molar concentrations, the relative height of the plateau observed in the case of divalent salt is larger compared with the case of monovalent salt. At 10 mM MgCl_2_, the height of the plateau is comparable with that observed for 1 M NaCl. In general, with MgCl_2_, the plateau extends down to low extensions (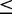
4 μm), falling to 0 force more steeply at low extensions. This effect is neat at high divalent salt conditions (Figure 4); in contrast, in the other extreme conditions, the equivalent FECs at low monovalent salt conditions for NaCl (e.g. 10 mM) hardly exhibit a plateau (Figure 3).

#### Analysis with the WLC

To quantify the elastic properties of ssDNA, we fitted the FECs to the WLC using the Marko–Siggia interpolation formula (28):
(2)
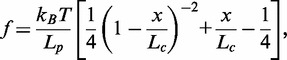

where *L_p_* and *L_c_* are the persistence and contour lengths, respectively. Even if our molecular construct contains a short stretch of dsDNA (88 bp) serially linked to the ssDNA, the contribution of these dsDNA parts to the measured elastic response of the ssDNA is expected to be negligible (<1%) due to its much higher stiffness (50-fold per base pair) and much shorter length. FECs have been fitted only between 10 and 40 pN to avoid the presence of secondary structure. In fact, secondary structure formation should be suppressed at forces >10–15 pN, large enough to unzip DNA (36). In Tables 1 and 2, we report the values of the persistence length, *L_p_*, and contour length, *L_c_*, obtained from our fits. For every salt concentration, we measured the response of ∼10 different molecules, and the value of the persistence length is the average of the results obtained over the three best curves where the effect of non-specific binding of the oligo concentration was found to be negligible (see also Supplementary Section S7).

**Table 1.** Persistence and contour lengths obtained from a fit of the FECs (in the range 10–40 pN) to the WLC model at varying NaCl concentrations

NaCl (mM)	*L_p_* (nm)	Standard deviation (nm)	*L_c_* (nm/base)	Standard deviation (nm/base)
10	1.19	0.06	0.68	0.02
25	1.04	0.06	0.69	0.02
50	0.99	0.05	0.68	0.02
100	0.87	0.04	0.69	0.03
250	0.81	0.04	0.69	0.03
500	0.78	0.04	0.69	0.02
1000	0.76	0.05	0.70	0.02

A fixed amount of 9 mM of monovalent ion concentration from the buffer must be added at each condition.

**Table 2.** Persistence length and contour length obtained from a fit of the FECs (in the range 10–40 pN) to the WLC model at varying MgCl_2_ concentrations

MgCl_2_ (mM)	*L_p_* (nm)	Standard deviation (nm)	*L_c_* (nm/base)	Standard error (nm/base)
0.5	0.93	0.06	0.69	0.03
1	0.86	0.04	0.69	0.02
2	0.78	0.04	0.70	0.02
4	0.79	0.05	0.70	0.02
10	0.75	0.04	0.70	0.02

**Figure 5.**
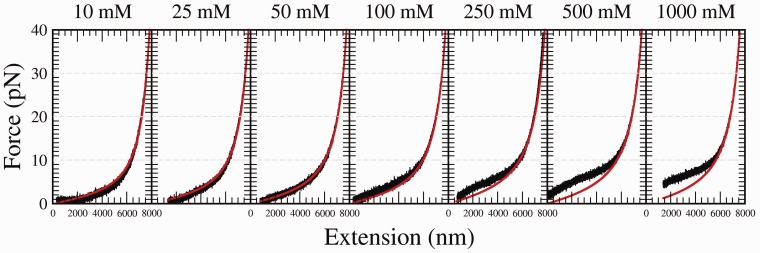
Comparison between an experimental FEC (black) and a fit to the WLC model in the range 10–40 pN (red/gray) at different NaCl concentrations. Results are shown for a representative molecule.

**Figure 6.**
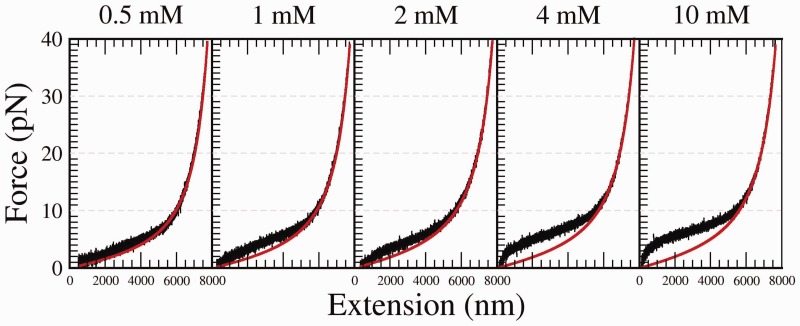
Comparison between an experimental FEC (black) and a fit to the WLC model in the range 10–40 pN (red/gray) at different MgCl_2_ concentrations. Results are shown for a representative molecule.

In Figures 5 and 6, experimental FECs fitted to the WLC model in the range 10–40 pN are shown for each concentration of NaCl and MgCl_2_, respectively. The WLC model describes well the curves in the range of the fit, but fails to describe the FEC at forces <10 pN. In particular, at these forces, positive force deviations from the WLC model are observed at concentrations that are >100 mM for monovalent salt and in the whole range of concentrations for divalent salt. Such deviations are due to secondary structure formation. Moreover, at monovalent salt concentrations that are <50 mM, some negative deviations at forces <10 pN are also observed. These can be attributed to excluded volume effects (18,20,21). In fact, at low concentrations, the Debye length can be larger than the persistence length of ssDNA so electrostatic repulsion between different DNA segments inhibits the bending of the filament.

One may ask whether bond extensibility [not included in the WLC formula Equation (2)] could play a role in this force range (10–40 pN). To answer this, we have fit the data with the extensible version of the WLC (28). From this analysis, we could not appreciate a significant improvement of the quality of the fits, and the values of the fitting parameters changed <3% for *L_p_* and <1% for *L_c_* suggesting that at force <40 pN, the inextensible WLC model is sufficient to describe the observed elastic behavior.

#### Behavior of the persistence length at different ionic conditions

The persistence length, *L_p_*, for a semi-flexible polymer can be written as
(3)
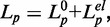

where 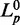 is the intrinsic persistence length and 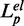 is an electrostatic contribution that depends on the Debye screening length and consequently on the inverse of the square root concentration of ions in solution (37–41). This behavior has been recently tested (42–44) with the electrostatic term, 
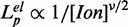
, where the exponent ν can take different values: 2 [as predicted by the Odijk–Skolnick–Fixman theory (37,38)], 1 [as obtained by variational methods (45,46)] or even <1 (47).

**Figure 7.**
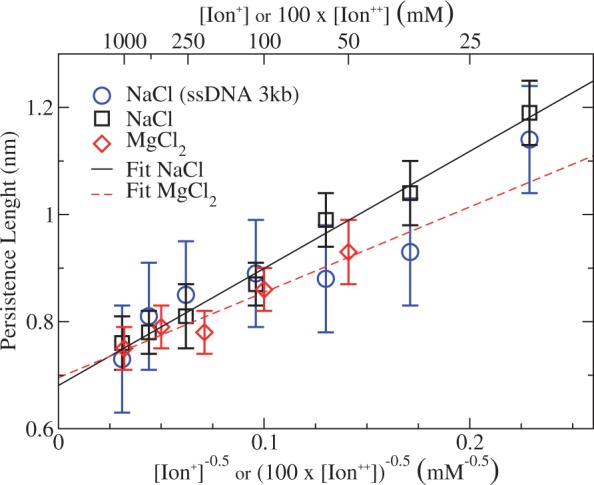
Persistence length values for ssDNA at different salt conditions in NaCl (black squares) and MgCl_2_ (red diamonds). In blue circles, we show data obtained from (25). Magnesium concentrations along the x-axis have been multiplied by a factor 100. Solid black and dashed red lines correspond to the linear fits to NaCl and MgCl_2_ data. The parameters obtained from the fit 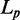
=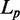
(0)+A* 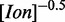 are: 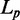
(0) = 0.68 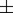 0.04 and A = 2.19 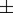 0.10 (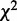 = 0.666) in the case of NaCl; and 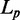
(0)=0.70 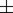 0.04 and A = 1.59 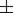 0.57 (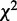
=0.701) in the case of MgCl_2_.

Figure 7 shows *L_p_* as a function of the inverse of the squared root of salt concentration. Our results are in agreement with previous measurements obtained by chemical denaturation of a 3-kb dsDNA (Figure 7, blue circles), albeit the present method provides significantly lower standard errors. In Supplementary Section S4, we also compare the curves of force versus relative extension in both cases (25). The good agreement found at high forces between both measurements shows that the force is a homogeneous function of the relative extension, a unique feature of ideal elastic models. The disagreement observed at 1 M NaCl (Supplementary Information Figure S3, right) shows that secondary structure formation starts to be important at forces as high as 15 pN.

The comparison between monovalent and divalent salts shown in Figure 7 shows a linear dependence for both types of salts that collapse into a single linear trend if a factor of 100 between both ions is considered (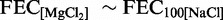). These results confirm the hypothesis that magnesium is ∼100 times more efficient than sodium in non-specifically screening DNA, which is reflected in the elastic properties of ssDNA. Moreover, this effect hinders the presence of monovalent ions due to the protonation of Tris in the solutions with magnesium. As has been suggested in the literature (22), such factor between monovalent and divalent salt concentrations cannot be explained by a Debye–Hückel picture of a diffuse cloud of screening counterions. However using strongly correlated liquid models of counterion/macroions interaction (48) a factor ranging from 50 to 200 is expected (22). Our results are consistent with findings reported in the case of the folding of a ribozyme (49) and in pulling experiments of RNA hairpins (43,44).

Figure 7 also shows the results of a fit with ν = 1. For both salts, a value of 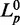 equal to 0.7 nm is obtained. This value is in good agreement with the previously reported values for single-stranded nucleic acids, in particular for poly-U (0.67 nm) (14,50) and for chemically denatured ssDNA (0.6 nm) (20,21). If we perform a fit with ν, a fitting parameter, we then obtain ν = 1.15 ± 0.25 in the case of NaCl and ν = 1.65 ± 0.85 in the case of MgCl_2_.

#### Analysis with the Ex-FJC

**Figure 8.**
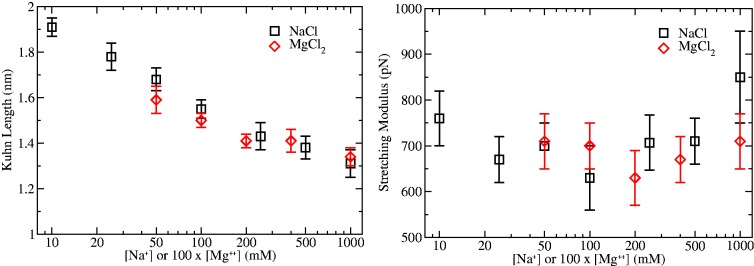
Kuhn length and stretching modulus values for ssDNA versus salt concentration (normal-log scale) for NaCl (black squares) and MgCl_2_ (red diamonds). Magnesium concentrations have been multiplied by a factor 100.

Another commonly used model to describe the FEC of ssDNA is the Ex-FJC model (29):
(4)
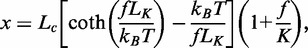

where *L_K_* is the Kuhn length, the dimension of the minimal segment on which the polymer can be mapped, and *K* is the stretching modulus. Similarly to (16), fits of the FECs were performed by fixing the contour length of the molecule to the crystallographic length *l* of ssDNA as described in (18) (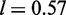
nm or *L_c_* = 7857 nm for a 13650 bases molecule). The fits were carried out in the range 10–40 pN (Supplementary Section S5), and the results are reported in Tables 3 and 4 and summarized in Figure 8. The Kuhn length decreases as the salt concentration increases with a logarithmic dependence on ionic strength. Our values for the Kuhn length agree with those reported in the literature, which fall in the range 1.5–1.6 nm at [NaCl] = 150 mM (16,51,52) and in phosphate-buffered saline (53). Let us note that, for a fixed value of the contour length of the ssDNA, the introduction of extensibility in Equation (4) through the stretching modulus *K* implies to use a slightly shorter length of the crystallographic value (0.57 nm) with respect to the experimentally reported range (0.60–0.70 nm). *K* shows a non-linear trend with concentration, and ranges between 650 and 850 pN. In the literature, values of 800 pN at [NaCl] = 150 mM (16,51,52) and 300 pN in phosphate-buffered saline (where monovalent salt concentration is ∼150mM) (53) have been reported. The values for *L_K_* given in Tables 3 and 4 and those of the persistence length *L_p_* shown in Tables 1 and 2 approximately follow the relation 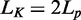 as expected for semiflexible polymer models when the value of *L_p_* is comparable with the segment length *l*.

**Table 3.** Kuhn length *L_K_* and stretch modulus *K* obtained by fitting FECs in the range 10–40 pN at varying NaCl concentrations

NaCl (mM)	*L_K_* (nm)	Standard deviation (nm)	*K* (pN)	Standard deviation (pN)
10	1.91	0.04	760	50
25	1.78	0.06	670	50
50	1.68	0.05	700	50
100	1.55	0.04	630	70
250	1.43	0.06	707	60
500	1.38	0.05	710	50
1000	1.31	0.06	850	100

A fixed amount of 9 mM of monovalent ion concentration contributed by the buffer must be added at each condition.

**Table 4.** Kuhn length *L_K_* and stretch modulus *K* obtained by fitting FECs in the range 10–40 pN at varying MgCl_2_ concentrations

MgCl_2_ (mM)	*L_K_* (nm)	Standard deviation (nm)	*K* (pN)	Standard deviation (pN)
0.5	1.59	0.06	710	60
1	1.50	0.03	700	50
2	1.41	0.03	630	60
4	1.41	0.05	670	50
10	1.34	0.04	710	60

#### Low salt conditions: indications on effective thickness

**Figure 9.**
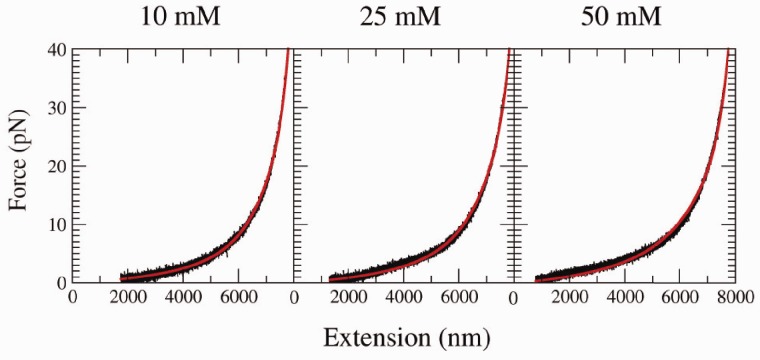
Fit of FECs to the TC model at low monovalent salt conditions. Experimental data (black) and fits to the TC model (red/gray) for low NaCl concentrations.

To analyze the effect of the excluded volume, FECs at low monovalent salt concentration (10–50 mM of NaCl, where secondary structure formation is not observed) have been fitted to the TC model (31,32,42) in the whole range of forces studied. This model takes into account excluded volume effects for a polymer, and it can be used to infer the effective thickness of polyelectrolytes from stretching measurements. In the TC model, the polymer is described as a tube of uniform cross-section Δ, with minimal segments of length *l*, persistence length *L_p_* and contour length *L_c_*. It provides a relation between the force applied *f* and the extension of the polymer *x*. This model has been used to describe the FEC of a homopolymeric nucleic acid Poly(U) (32). Figure 9 shows fits to the FEC, while Table 5 reports the values of the fits. The values obtained are again compatible with those reported for homopolymers in the same salt concentration range [Poly-U, Δ in the range 0.8–0.9 nm (32)]. Our results show that the effective thickness decreases with salt concentration owing to the more effective screening of the phosphate chain by cations.

**Table 5.** Geometric parameters obtained by fitting the FECs at low monovalent salt concentration with the TC model

NaCl (mM)	Δ (nm)	St.Dv. (nm)	*L*(nm)	St.Dv. (nm)	*L_c_* (μm)	St.Dv. μm
10	0.83	0.03	1.32	0.07	8.48	0.10
25	0.74	0.04	1.21	0.06	8.57	0.10
50	0.71	0.02	1.27	0.03	8.46	0.10

A fixed amount of 9 mM of monovalent salt concentration contributed by the buffer must be added at each condition.

### Secondary structure formation

The blocking oligonucleotide used to inhibit the formation of the hairpin does not prevent the formation of non-native secondary structure at low enough forces driven by stacking and base pairing interactions. In fact, the shoulder observed in the FECs at high salt concentrations suggests that ssDNA collapses in a condensed phase. This effect is more prominent in the presence of divalent cations indicating the successful coordinated action of the doubly charged cations to bring distal segments of ssDNA close to each other. In fact, stretching/releasing curves of ssDNA in the presence of MgCl_2_ exhibit larger hysteresis effects than for NaCl (data not shown), associated to the formation and rupture of secondary structure.

Despite the fact that we do not have a phenomenological model describing this condensed phase, the large number of structural motifs that can be formed and their low energetic stability produce a soft shoulder in the FEC without any visible force rips. The competition between the large number of possible configurations that can be formed in such condensed phase suggests that configurational entropy (rather than enthalpy) is the main driving force toward the formation of secondary structure that prevents the reformation of the native hairpin. Several effects contribute to the soft shoulder observed in the FEC: the large flexibility of the ssDNA due to its low persistence length, the so-called compensation effect (54,55) between different structural motifs that unfold/fold independently due to thermal forces and the low cooperativity of the unfolding/folding transition expected for such motifs. Next, we quantify the amount of secondary structure formed as a function of force over different salt conditions.

#### Estimation of the fraction of unpaired bases at high salt condition

**Figure 10.**
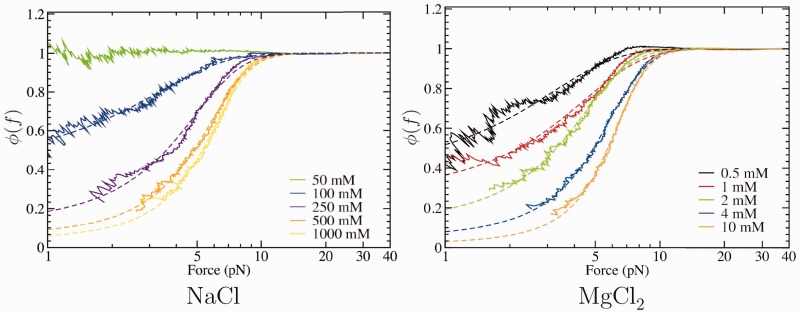
Fraction of unpaired ssDNA bases as a function of force. Dashed lines are fits to experimental data using Equation (5).

To have a quantitative measure of the fraction of ssDNA that forms secondary structure, we used the WLC model to estimate the effective contour length 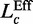 as a function of force. For each FEC, we extracted the effective contour length by fixing the value of the persistence length to that obtained by fitting the elastic response in the range 10–40 pN. If the WLC model were to describe well the whole range of measured forces, the effective contour length would be equal to the one measured in the force range 10–40 pN (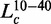) and should not depend on force even at low forces. The fraction of unpaired ssDNA bases is then simply given by the ratio 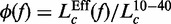, and results are shown in Figure 10.

Below 50 mM NaCl, 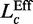 increases at forces <5 pN and the fraction 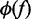 becomes >1 (data not shown). This odd result indicates the inadequacy of the WLC model in this regime and is related to excluded volume effects that become prominent at low salt concentration, as discussed in the previous section. Above 100 mM NaCl, and in all explored concentrations of divalent salt, 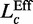 decreases with decreasing force. We attribute this feature to the formation of secondary structure. At low forces (<5 pN), the higher the salt concentration, the shorter the effective contour length, meaning that at high monovalent salt concentration (or in presence of divalent salt), the fraction of paired bases increases. At the lowest measured forces (1–2 pN), the fraction of unpaired bases can reach up to 10–20% at the highest salt concentrations (namely, 1 M of NaCl and 10 mM of MgCl_2_).

To extract the fraction of unpaired bases at 0 force, 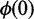, we fit data to the following phenomenological formula,
(5)
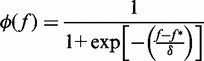

where 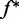 is the critical force at which half of the nucleotides are paired, while δ is a measure of the width of the force region in which ssDNA starts to form secondary structure. An analogous exponential dependence of the fraction of secondary structures has been suggested on the basis of mesoscopic models that take into account the formation of multiple and degenerate structures (56). Figure 10 shows fits of Equation (5) to the experimental data, and fitting parameters are summarized in Table 6.

**Table 6.** Parameters obtained from a fit to Equation (5) of the curves presented in Figure 10

NaCl (mM)	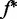 (pN)	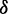 (pN)	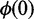
100	0.440	2.090	0.448
250	3.6	1.75	0.313
500	4.850	1.637	0.049
1000	5.322	1.520	0.029

MgCl_2_ (mM)	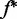 (pN)	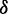 (pN)	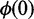

0.5	0.625	1.958	0.421
1	2.270	2.070	0.250
2	3.310	1.580	0.110
4	4.899	1.610	0.046
10	5.681	1.430	0.018

For the NaCl case a fixed amount of 9 mM of salt concentration from the buffer must be added at each condition.

**Figure 11.**
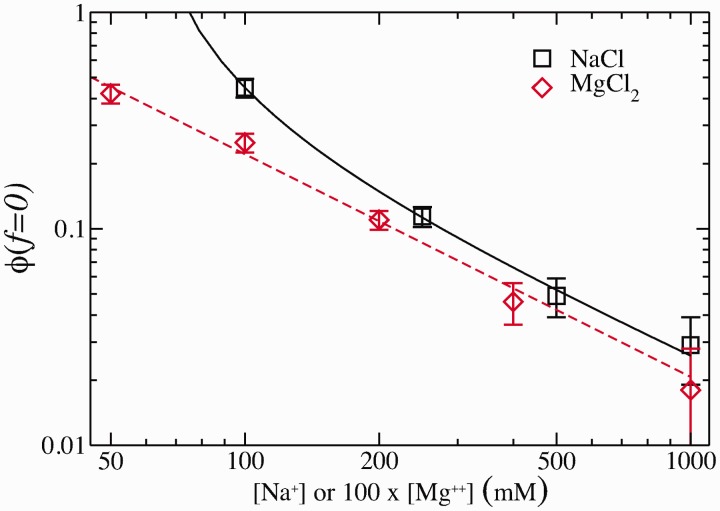
Fraction of the unpaired bases at 0 force 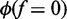 at different salt concentrations. Logarithmic scale is used for both axes. Black squares stand for NaCl and red diamonds stand for MgCl_2_. Magnesium concentrations have been multiplied by a factor 100. Lines are fits to the power law dependence, 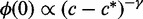 (see text for details).

For both monovalent and divalent ions, 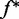 increases, whereas the fraction of unpaired bases at 0 force, 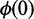, decreases with salt concentration, as expected. Figure 11 shows 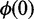 as a function of salt concentration. At both 1 M of NaCl and 10 mM of MgCl_2_, 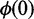 has almost reached the minimal value of 0, meaning that at 0 force and high salt, almost all bases contribute to secondary structure formation. The dependence of 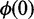 with salt concentration can be described by a power law 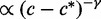 where 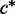 stands for a critical concentration and 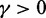 is an exponent. From the fit (Figure 11), one gets 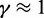 for both monovalent and divalent salts, but with a different value of 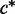 (65 mM and 0 mM for NaCl and MgCl_2_, respectively, see Supplementary Section S6). At high *c*, we find 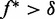, meaning that we can approximate 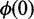 in Equation (5) by 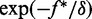 showing that 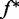 increases logarithmically with *c*, as indicated in the results of Table 6.

Finally, we also checked the effect of the blocking oligo at high salt condition (Supplementary Section S7). The oligo was designed to selectively attach to the loop; however, it also binds to other regions along the ssDNA. This is confirmed by the observed decrease of height of the force plateau when increasing the oligo concentration.

## CONCLUSIONS

In this article, we investigated the elastic properties and the formation of secondary structure in ssDNA using the approach of the blocking oligo. We systematically explored a range of different concentration of monovalent and divalent salts observing the formation of a plateau in the FEC of the ssDNA, which we interpret as evidence of secondary structure formation. At a given force, the amount of secondary structure (i.e. the fraction of paired bases) increases with salt concentration. Fitting the FECs of ssDNA to the inextensible WLC, we have found that the persistence length depends linearly with the inverse of the square root concentration of monovalent and divalent salts (31,32,40), the electrostatic contribution being apparently proportional to the Debye length. Interestingly enough, divalent cations have a notably greater effect in screening electrostatic charge than monovalent salt, as has been observed in previous thermal- and tension-induced base pair melting. The intrinsic persistence length is ∼0.7 nm for both types of salt, and the effectivity of divalent cations in screening electrostatic interactions appears to be 100-fold as compared with monovalent salt, in line with what has been recently reported for single-stranded RNA (43). The values of the Kuhn length obtained from the Ex-FJC model display a similar behavior. The contour length of the ssDNA shows a weak dependence with salt concentration, suggesting that the variation of the ionic force of the medium does not affect the conformation of the sugars in the force range explored; in fact, a fraction of the deoxyriboses could interconvert from C3-endo (interphosphate distance 0.6 nm) to C2-endo conformation (interphosphate distance 0.7 nm) resulting in a change in the contour length (2,3). The WLC is sufficient to describe phenomenologically the elastic behavior of ssDNA at forces <40 pN, but only if the contour length of the ssDNA is a fitting parameter to the elastic model. In contrast, if we fix the contour length of the ssDNA using the crystallographic interphosphate distance, then we must use an extensible model (such as the Ex-FJC) to accurately fit the FECs. We also reported an analysis of the data based on the TC model to extract the effective thickness of the ssDNA at low monovalent salt condition. Finally, we characterized the formation of secondary structures from the analysis of the FECs taken at high salt condition and proposed a phenomenological formula quantifying the amount of non-native secondary structure.

All these behaviors rely on a trade-off between filament flexibility and electrostatics. Electrostatic repulsion between phosphate groups along the backbone stiffens the filament. On increasing salt concentration, the backbone self-repulsion is screened and hence its persistence length decreases. Moreover, the higher the salt, the larger the screening of the backbone, facilitating base pair formation. Overall, these effects contribute to the formation of non-native secondary structure.

As a further step, it would be interesting to apply our technique to study the FEC in the presence of other kind of cations, both monovalent and divalent, such as potassium and calcium, or even multivalent cations, such as hexamine cobalt compounds and polyamines. Finally, a deeper understanding of the kinetics and thermodynamics of non-specific secondary structure formation could be obtained by applying the present methodology to investigate ssDNA molecules of varying length and base pair composition.

## SUPPLEMENTARY DATA

Supplementary Data is available at NAR Online.

## FUNDING

Erasmus Placement grant [KTEU EP 4 to A.B.]; Associazione Italiana Ricerca Cancro [AIRC 5 per mille No. 12214 to A.B.]; Institucio Catalana de Recerca i Estudis Avancats [Academia 2008 to J.C.S., Academia Award 2008 to F.R.]; Human Frontier Science Program [RGP55-2008 to F.R.]; Spanish Ministry of Economy and Competitiveness [FIS2010-19342 to F.R.]. Funding for open access charge: European Union Seventh Framework Programme (FP7/2007-2013) under grant agreement [n° 308850].

*Conflict of interest statement*. None declared.

## Supplementary Material

Supplementary Data

## References

[1] Liang X, Kuhn H, Frank-Kamenetskii MD (2006). Monitoring single-stranded DNA secondary structure formation by determining the topological state of DNA catenanes. Biophys. J..

[2] Saenger W (1984). Principles of Nucleic Acid Structure.

[3] Bloomfield VA, Crothers DM, Tinoco IJ (1999). Nucleic Acids.

[4] Zhang Y, Zhou H, Ou-Yang Z-C (2001). Stretching single stranded DNA: interplay of electrostatic, base-pairing and base-pair stacking interactions. Biophys. J..

[5] Zhou H, Zhang Y (2001). Pulling hairpinned polynucleotide chains: does base-pair stacking interaction matter?. J. Chem. Phys..

[6] Seol Y, Skinner GM, Visscher K (2007). Stretching of homopolymeric RNA reveals single-stranded helices and base-stacking. Phys. Rev. Lett..

[7] Ke C, Humeniuk M, S-Gracz H, Marszalek PE (2007). Direct measurement of base stacking interactions in DNA by single-molecule atomic-force spectroscopy. Phys. Rev. Lett..

[8] Ehresmann C, Bauldin F, Mougel M, Romby P, Ebel JP, Ehresmann B (1987). Probing the structure of RNA. Nucleic Acids Res..

[9] Dong F, Allawi HT, Anderson T, Neri BP, Lyamichev VI (2001). Secondary structure prediction and structure specific sequence analysis of single stranded DNA. Nucleic Acids Res..

[10] Ritort F (2006). Single-molecule experiments in biological physics: methods and applications. J. Physics: Condensed Matter.

[11] Kumar S, Mishra G (2011). Stretching single stranded DNA. Soft Matter.

[12] Lacy MJ, Voss EW (1989). Direct adsorption of ssDNA to polystyrene for characterization of the DNA/anti-DNA interaction, and immunoassay for anti-DNA autoantibody in New Zealand White mice. J. Immunol. Methods.

[13] Manohar S, Mantz AR, Bancroft KE, Hui C-Y, Jagota A, Vezenov DV (2008). Peeling single-stranded DNA from graphite surface to determine oligonucleotide binding energy by force spectroscopy. Nano Lett..

[14] Petrovykh DY, Kimura-Suda H, Whitman LJ, Tarlov MJ (2003). Quantitative analysis and characterization of DNA immobilized on gold. J. Am. Chem. Soc..

[15] Petrovykh DY, rez Dieste VP, Opdahl A, Kimura-Suda H, Sullivan JM, Tarlov MJ, Himpsel FJ, Whitman LJ (2006). Nucleobase orientation and ordering in films of single-stranded DNA on gold. J. Am. Chem. Soc..

[16] Smith SB, Cui Y, Bustamante C (1996). Overstretching B-DNA: the elastic response of individual double-stranded and single-stranded DNA molecules. Science.

[17] Candelli A, Hoekstra T, Farge G, Gross P, Peterman EG, Wuite GL (2013). A toolbox for generating single-stranded DNA in optical tweezers experiments. Biopolymers.

[18] Dessinges M-N, Maier B, Zhang Y, Peliti M, Bensimon D, Croquette V (2002). Stretching single stranded DNA, a model polyelectrolyte. Phys. Rev. Lett..

[19] Montanari A, Mézard M (2001). Hairpin formation and elongation of biomolecules. Phys. Rev. Lett..

[20] Saleh OA, McIntosh DB, Pincus P, Ribeck N (2009). Nonlinear low-force elasticity of single-stranded DNA molecules. Phys. Rev. Lett..

[21] McIntosh DB, Ribeck N, Saleh OA (2009). Detailed scaling analysis of low-force polyelectrolyte elasticity. Phys. Rev. E.

[22] McIntosh DB, Saleh OA (2011). Salt species-dependent electrostatic effects on ssDNA elasticity. Macromolecules.

[23] Manosas M, Xi XG, Bensimon D, Croquette V (2010). Active and passive mechanisms of helicases. Nucleie Acids Res..

[24] Camunas-Soler J, Frutos S, Bizarro CV, de Lorenzo S, Fuentes-Perez ME, Ramsch R, Vilchez S, Solans C, Moreno-Herrero F, Albericio F (2013). Electrostatic binding and hydrophobic collapse of peptide nucleic acid aggregates quantified using force spectroscopy. ACS Nano.

[25] Huguet JM, Bizarro CV, Forns N, Smith SB, Bustamante C, Ritort F (2010). Single-molecule derivation of salt dependent base-pair free energies in DNA. Proc. Natl Acad. Sci. USA.

[26] Bustamante C, Smith SB, Liphardt J, Smith D (2000). Single-molecule studies of DNA mechanics. Curr. Opin. Struct. Biol..

[27] Bustamante C, Marko JF, Siggia ED, Smith SB (1994). Entropic elasticity of lambda-phage DNA. Science.

[28] Marko JF, Siggia ED (1995). Stretching DNA. Macromolecules.

[29] Smith SB, Finzi L, Bustamante C (1992). Direct mechanical measurements of the elasticity of single DNA molecules by using magnetic beads. Science.

[30] Johnson DS, Bai L, Smith BY, Patel SS, Wang MD (2007). Single molecule studies reveal dynamics of DNA unwinding by ring-shaped T7 helicase. Cell.

[31] Marenduzzo D, Micheletti C (2003). Thermodynamics of DNA packaging inside a viral capsid: the role of DNA intrinsic thickness. J. Mol. Biol..

[32] Toan NM, Marenduzzo D, Micheletti C (2005). Inferring the diameter of a biopolymer from its stretching response. Biophys. J..

[33] Smith SB, Cui Y, Bustamante C (2003). Optical-trap force transducer that operates by direct measurement of light momentum. Methods Enzymol..

[34] Forns N, de Lorenzo S, Manosas M, Hayashi K, Huguet J, Ritort F (2011). Improving signal/noise resolution in single molecules experiments using molecular constructs with short handles. Biophys. J..

[35] Wolfe AR, Meehan T (1994). The effect of sodium ion concentration on intrastrand base-pairing in single-stranded DNA. Nucleic Acids Res..

[36] Essevaz-Roulet B, Bockelmann U, Heslot F (1997). Mechanical separation of the complementary strands of DNA. Proc. Natl Acad. Sci. USA.

[37] Odijk T (1977). Polyelectrolytes near the rod limit. J. Polym. Sci. Polym. Phys. Ed..

[38] Skolnick J, Fixman M (1977). Electrostatic persistence length of a wormlike polyelectrolyte. Macromolecules.

[39] Manning GS (2001). Counterion condensation on a helical charge lattice. Macromolecules.

[40] Caliskan G, Hyeon C, Perez-Salas U, Briber RM, Woodson SA, Thirumalai D (2005). Persistence length changes dramatically as RNA folds. Phys. Rev. Lett..

[41] Toan NM, Thirumalai D (2010). Theory of biopolymer stretching at high forces. Macromolecules.

[42] Toan NM, Micheletti C (2006). Inferring the effective thickness of polyelectrolytes from stretching measurements at various ionic strengths: applications to DNA and RNA. J. Phys. Condens. Matter.

[43] Bizarro CV, Alemany A, Ritort F (2012). Non specific binding of Na^+^ and Mg_2_^+^ to RNA determined by force spectroscopy methods. Nucleic Acids Res..

[44] Chen H, Meisburger SP, Pabit SA, Sutton JL, Webb WW, Pollack L (2012). Ionic strenght-dependent persistence lenghts of single stranded RNA and DNA. Proc. Natl Acad. Sci. USA.

[45] Barrat J-L, Joanny J-F (1993). Persistence length of polyelectrolyte chains. Europhys. Lett..

[46] Ha BY, Thirurmalai D (1995). A mean-field model for semiflexible chains. J. Chem. Phys..

[47] Micka U, Kremer K (1996). Persistence length of the Debye Hueckel model of weakly charged flexible polyelectrolyte chains. Phys. Rev. E.

[48] Shklovskii BI (1999). Screening of a macroion by multivalent ions: correlation-induced inversion of charge. Phys. Rev. E.

[49] Heilman-Miller SL, Thirumalai D, Woodson SA (2001). Role of counterion condensation in folding of the tetrahymena ribozyme. I. equilibrium stabilization by cations. J. Mol. Biol..

[50] Seol Y, Skinner G, Visscher K (2004). Elastic properties of a single-stranded charged homopolymeric ribonucleotide. Phys. Rev. Lett..

[51] Rief M, Clausen-Schaumann H, Gaub HE (1999). Sequence-dependent mechanics of single DNA molecules. Nat. Struct. Biol..

[52] Clausen-Schaumann H, Rief M, Tolksdorf C, Gaub HE (2000). Mechanical stability of single DNA molecules. Biophys. J..

[53] Danilowicz C, Lee CH, Coljee VW, Prentiss M (2007). Effects of temperature on the mechanical properties of single stranded DNA. Phys. Rev. E.

[54] Gerland U, Bundschuh R, Hwa T (2001). Force-induced denaturation of RNA. Biophys. J..

[55] Gerland U, Bundschuh R, Hwa T (2003). Mechanical probing the folding pathway of single RNA molecules. Biophys. J..

[56] Manosas M, Junier I, Ritort F (2008). Force-induced misfoldig in RNA. Phys. Rev. E.

