# Supplementary material for: Elastic properties and secondary structure formation of single-stranded DNA at monovalent and divalent salt conditions
Source: Nucleic Acids Res. 2013 Nov 12;42(3):2064–74. doi: 10.1093/nar/gkt1089 (PMC3919573; doi:10.1093/nar/gkt1089)
Supplement: Supplementary Data [file supp_42_3_2064__index.html]

Elastic properties and secondary structure formation of single-stranded DNA at monovalent and divalent salt conditions — Elastic properties and secondary structure formation of single-stranded DNA at monovalent and divalent salt conditions — Supplementary Data 

# Elastic properties and secondary structure formation of single-stranded DNA at monovalent and divalent salt conditions

## Supplementary Data

files

**Files in this Data Supplement:**

- Supplementary Data - pdf file
