# Supplementary material for: Elastic properties and secondary structure formation of single-stranded DNA at monovalent and divalent salt conditions
Source: Nucleic Acids Res. 2013 Nov 12;42(3):2064–74. doi: 10.1093/nar/gkt1089 (PMC3919573; doi:10.1093/nar/gkt1089)
Supplement: Supplementary Data [file supp_gkt1089_SuppInfo.pdf]

Supporting Information

S.1 Materials and molecular synthesis of the DNA hairpin

N6-methyladenine free λ-DNA (New England Biolabs) is digested with BamHI (New England Biolabs) and phosphorylated at its 5-ends (T4 polynucleotide kinase, New England Bio- labs). The fragment of the dsDNA between positions 41733 and 48502 (the cosR end) is used as the stem of the DNA hairpin. Several oligonucleotides (sequences are reported in Table 1) are used as building blocks for the formation of the hairpin. The tetraloop that completes the hairpin is created using BamHI-loopII purchased from Eurons MWG Operon. This oligonucleotide forms a small hairpin structure whose stem has an overhang complementary to the BamHI restriction site. The dsDNA handles are formed from two oligonucletides (cosRlong and cosRshort from Sigma-Aldrich). The partial complementarity of the two oligos allows for the formation a protruding end complementary to the cosR. The cosRshort is functionalized at the 5-end with a biotin and the cosRlong is tailed with multiple digoxigenins at its 3-end (DIG Oligonucleotide tailing Kit 2nd Generation,Roche). After this procedure, cosRlong is purified with the QIAquick Nucleotide Removal Kit (QIAGEN). The dsDNA handles are formed by the hybridization of the unpaired regions of cosRlong and cosRshort with a complementary oligonucleotide (splint). The self-assembly of the hairpin is carried out with a thermal treatment. All the oligonucleotides and the 6770 bp stem are annealed by incubation for 10 minutes at 70°C, followed by incubation for 10 minutes at 55°C and cooling down to room temperature. The formation of phosphodiester bonds between the oligonucleotides (ligation) is performed in an overnight reaction at 16°C (T4 DNA ligase, New England Biolabs). The molecules obtained are stored in a solution containing Tris.HCl 10 mM, EDTA 1 mM, and NaCl 10 mM with pH=7.5.

In table 1 the sequences used in the experiments are also reported : the Blocking Loop oligonucleotide, used to prevent the rezipping of the hairpins, and A-Forward, used to check the specificity of former oligonucleotide.

Table 1: Oligonucleotides used for the hairpin synthesis and experiments

Oligonucleotide	Sequence
BamHI-loopII	5'-GATCGCCAGTTCGCGTTCGCCAGCATCCG<br>ACTACGGATGCTGGCGAACGCGAACTGGC-3'
cosRlong	5'-Pho-GGGCGGCGACCTAAGATCTATTATATATGTG<br>TCTCTATTAGTTAGTGGTGGAAACACAGTGCCAGCGC-3'
cosRshort	5'-Bio-AGTTAGTGGTGGAAACACAGTGCCAGCG<br>CAATAGAGACACATATATAATAGATCTT-3'
splint	5'-GCGCTGGCACTGTGTTTCCACCACTAACT-3'
Blocking Loop	5-TAGTCGGATGCTGGCGAACGCGAACTGGCG 3
A-Forward	5'-GGAATTCCGACTGGTGAGTACTCAACCAAGTC-3'

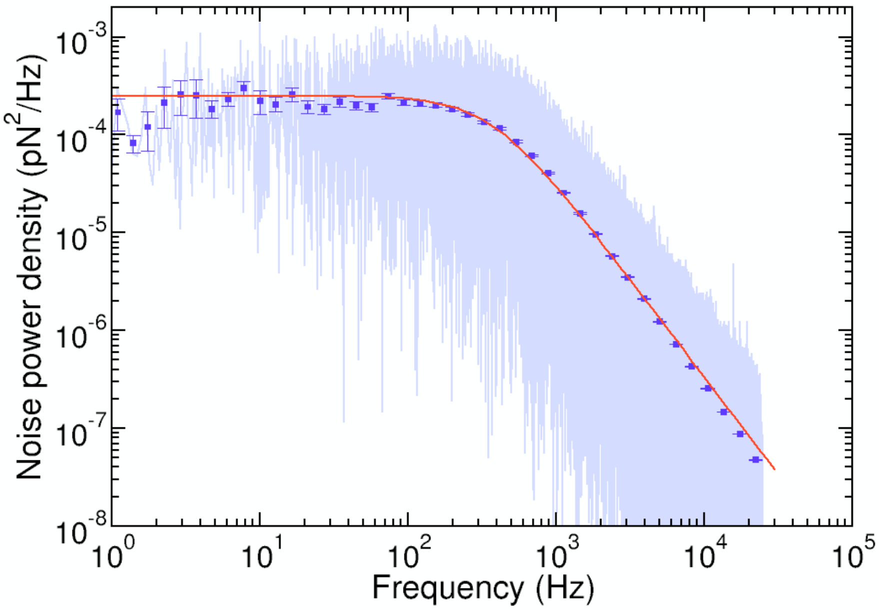

Figure 1: Power spectral density distribution of force fluctuations using a bead sized 3 μm i diameter. Ten seconds of output was recorded at 50 kHz. An exponential average is shown in dark blue, and a fit to eq. 1 in red.

S.2 Measurement of trap stiffness

The trap stiffness has been determined by recording the Brownian motion of the trapped bead at 50 kHz with a high-frequency data acquisition system directly connected to the electronic board of the optical tweezers as described in [1]. The power spectrum of the force fluctuations is calculated using an FFT algorithm, and fitted to the following Lorentzian:

$$< \Delta F^2(\omega) >= \frac{2k_B T k^2}{\gamma(w_c^2 + w^2)} \qquad (1)$$

where k is the trap stiffness, γ is the drag coefficient, and w_c is the corner frequency ($w_c = k/\gamma$). The fits are performed using a Levenberg-Marquadt algorithm and using as free-parameters k and γ. Using this method the trap stiffness was found to be 0.07 ± 0.005 pN/nm.

S.3 Unzipping of hairpins

Here we report the unzipping pattern obtained from the experiments both in monovalent and in divalent salt conditions. Figure 2 shows the unzipping pat-

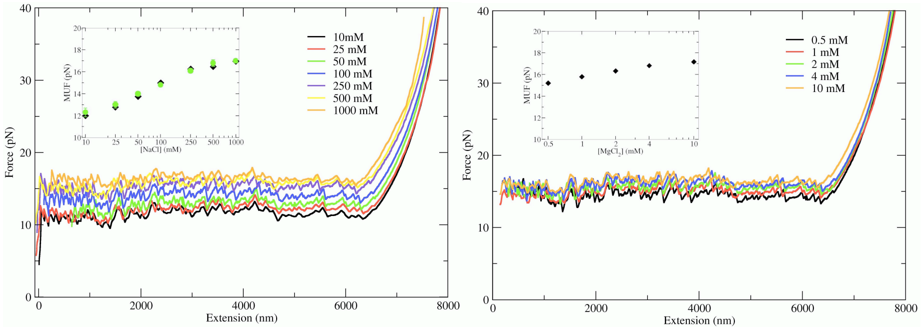

(a) NaCl (b) MgCl

Figure 2: Unzipping the molecular construct by varying monovalent and divalent salt concentrations. In the inset the mean unzipping force of the hairpins is reported (black diamonds). In the case of NaCl, the comparison with data of [2] (green circles) is also reported

tern for one molecule in the case of NaCl and $MgCl_2$.

The sawtooth patterns of this hairpin has been characterized previously [2]. For the case of monovalent salt, the mean unzipping force is measured and compared with results obtained from the literature in order to have a control on the synthesis of the molecules. The mean unzipping force is obtained by averaging force data along the range between 1000 and 6000 nm. As shown in the inset of Figure 2, our data agrees with previously published data in NaCl solutions [2]. For the results in $MgCl_2$ there are no data from literature to which we can compare ours. However the pattern of unzipping is almost identical to that found in the case of NaCl and the mean unzipping force increases increases divalent salt concentration, as expected.

S.4 Comparison with previously published force-extension curves of ssDNA

Here we present also the comparison between our force-extension curves of ssDNA and those reported in [2]. The results presented in that paper were obtained with a different molecular construct and protocol than ours. The instrumental setup is the same used here, but the molecule is a single strand obtained from a 3000 bps dsDNA. One of the two strands in the dsDNA is functionalized with biotin at 5' end and with digoxigenin at 3' end. The protocol for the attachment of the construct to the beads is similar but a chemical denaturation with NaOH is carried out. In this way a single strand of ssDNA is obtained for measurements. It must be stressed that this technique presents several technical difficulties because the second connection in the chamber is always hard to make. ssDNA in fact tends to non-specifically adsorb onto the surface of the beads and fishing a tether requires a longer number of trials.

In order to perform the comparison between the FECs obtained from the hairpin with the oligo and the molecule of 3000 bps, the molecular extension has been normalized by the contour length obtained by fitting data to the WLC at forces higher than 10 pN. Figure 3 shows this comparison. The qualitative agreement between the curves is quite remarkable at low salt condition (10-100 mM NaCl). At 1M NaCl concentration there is also agreement at forces higher than 15pN. Below that threshold, the behavior is quite different. A possible explanation to this discrepancy can be attributed either to cooperativity effects or difference in sequences that both lead to distinct secondory structure formation for the two molecular constructs.

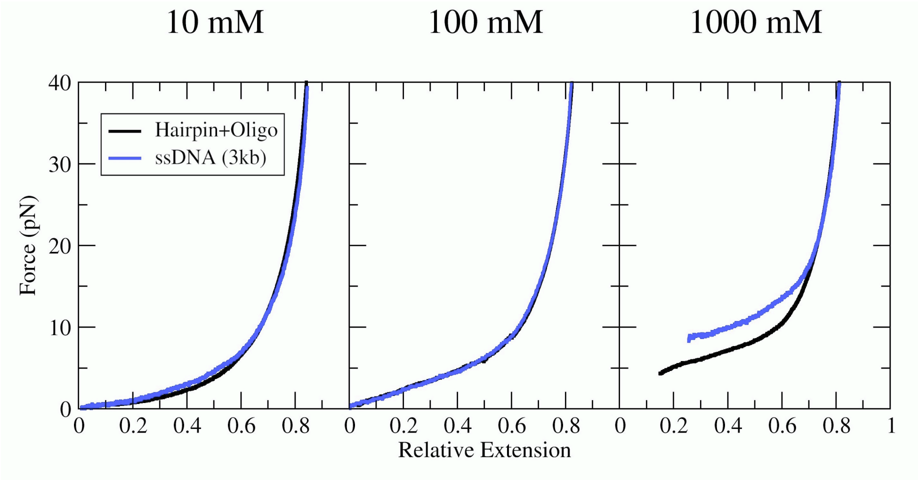

Figure 3: Comparison of FECs by plotting force versus relative extension (molecular extension normalized to the contour length) of data obtained with the method described here (black) and data reported in [2] for a ssDNA of 3 kbs (blue)

S.5 Comparison with the FJC

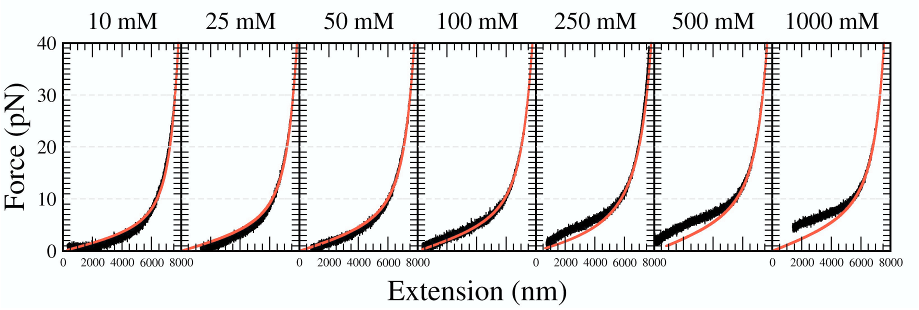

Figure 4: Comparison between the fit with the extensible freely jointed chain (Ex-FJC) model of the experimental FECs in the range 10-40 pN (red) with data covering the whole measured range (black) at different NaCl concentrations. Results are shown for one selected molecule.

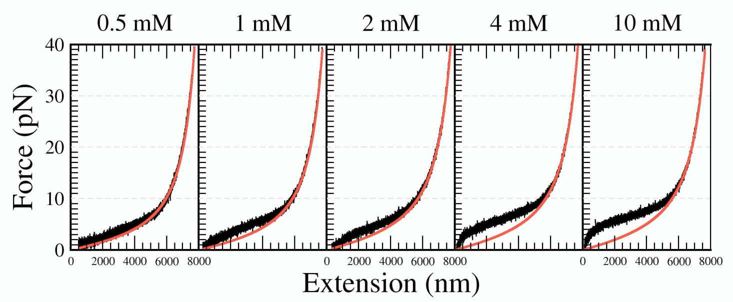

Figure 5: Comparison between the fit with the extensible freely jointed chain (Ex-FJC) model of experimental FECs in the range 10-40 pN (red) with data covering the whole measured range (black) at different $MgCl_2$ concentrations. Results are shown for one selected molecule.

S.6 Fitting the fraction of unpaired bases at different salt concentration

The formula used for the fit is

$$\phi(f=0) = a(c - c^*)^\gamma \quad . \tag{2}$$

By fixing $c^* > 0$ one finds: a=15, c^*=65 and γ=-0.93 for NaCl; and a=25.3, c^*=0.01 and γ=-1.03 for $MgCl_2$.

S.7 Effect of the oligo concentration

It is important to take into account, that the blocking oligonucleotide can also non-specifically bind along the whole molecule. This binding is undesirable as it could create short segments of dsDNA that interfere with the pure elastic response of ssDNA and with the random nonspecific secondary structure formation along the ssDNA filament. This effect is maximal at high ionic strengths due to the increased stability of duplex DNA. A way to minimize nonspecific binding is to use a very low concentration of blocking oligo, however this also increases the time required to obtain a ssDNA tether due to specific binding making experiments unpractical. Therefore to avoid this effect for measuring the ssDNA FECs we used the “flushing oligo” approach where the oligo is transiently flushed into the fluidics chamber. However, in order observe the effect of blocking oligo concentration on the FECs we performed a set of experiments at high salt condition (1M NaCl) with a fixed oligo concentration inside the microfluidic chamber.

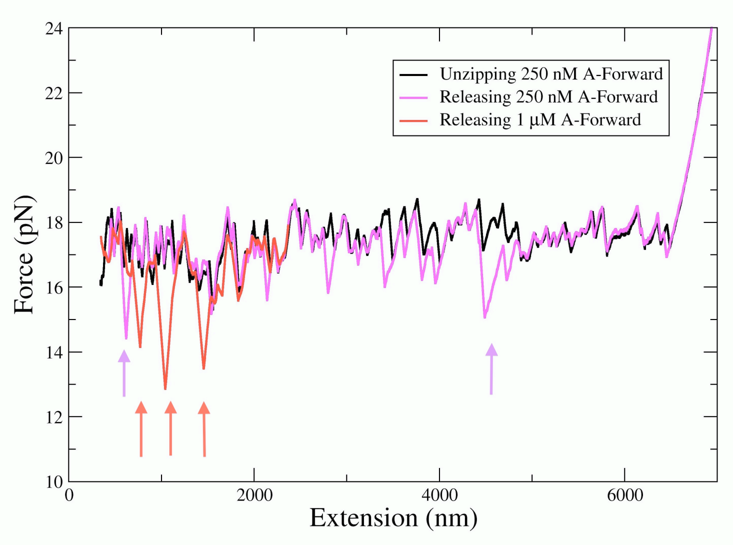

Figure 6: Effect of oligo A-forward (32nt) in the rezipping of the molecular construct at 1 M NaCl. The vertical arrows show the significative variation observed in the rezipping pattern for the case of 250 nM (violet) and 1 μM (red).

Before doing that, we also checked the binding specificity of the blocking oligo using a 32 bases oligo which is not complementary to any region of the DNA hairpin (A-Forward, Section S.1) at a concentration of 250 nM as a substitute of the blocking oligo. After unzipping the hairpin, when approaching the beads we observed a rezipping pattern in the force signal (Fig. 6) which is not identical to the one observed in the absence of oligo. In fact in some regions the hairpin

does not seem to fully rezip, probably due to the fact that the oligo binds to the filament preventing full rezipping. The same experiment carried out at 1 μM reveals a more prominent effect (Fig. 6). However in both cases the binding of the oligo to the filament is not stable and after a few seconds the oligo unbinds, and the typical full unzipping FEC measured in the absence of oligo is recovered (See green line, Figure 2, main text).

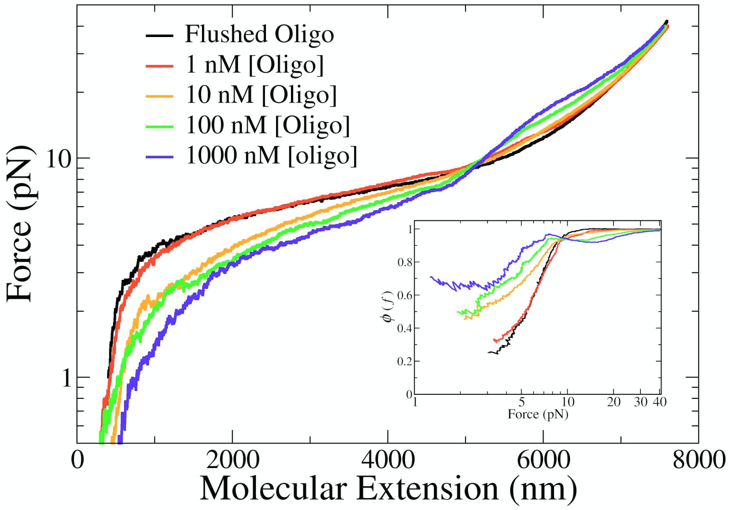

Figure 7: Experiments with fixed oligo concentration in TE 1M NaCl buffer solution (colored lines) compared with one curve (black line) obtained after a transient flush of a solution 50nM loop-complementary oligonucleotide. In the inset, we show the value of the fraction of unpaired ssDNA bases extracted from the effective contour length measurements for the same data.

In order to check if the blocking oligo concentration could affect the measurement of FECs by competing with the formation of secondary structure, we titrated the oligo concentration at the aforementioned high salt condition (1M NaCl). We performed experiments by varying blocking oligo concentration (Figure 7), observing a variation in the release pattern of the FECs as the oligo concentration increased. The experimental FECs at different oligo concentrations demonstrate that the experimental protocol described in the main text ("Flushing oligo" approach) yields effective oligo concentrations that are lower than 1 nM. By increasing the concentration of the oligo leads to a decrease of the plateau in the FECs at low forces. Apparently the oligo binds to sites that are randomly distributed along the sequence directly competing with the hybridization of complementary regions along the ssDNA. Interestingly, for higher oligo concentrations, not only the formation of secondary structure is reduced but also the shape of the FEC at high forces (f$\geq$10 pN) changes, maybe due to the shortening of the filament upon oligo binding.

By using the analysis described in the previous section, we have extracted the fraction of unpaired bases at different oligo concentrations (Figure 7, inset). In all cases we used the value of the persistence length obtained by flushing the oligo (Table 1, main text). At low oligo concentration (1 nM), the FEC slightly differs from the case in which the oligo is flushed into the chamber (protocol described in the main text).

However as the oligo concentration increases we find a systematic decrease in the effective contour length of the fiber and hence in the fraction of unpaired bases along the whole range of forces. This feature is probably due to the non-specific binding of oligonucleotides to the filament, that cause a decrease in its contour length. Non-specific binding of the oligo sequesters DNA segments thereby reducing secondary structure formation. This fact explains the trend shown in Figure 7 (inset) where the decrease observed in $\phi(f)$ at low forces (below 10 pN) is steepest when the oligo is flushed and, for the rest of cases, the steepness of the response at low extensions (i.e. preceding the force plateau) decreases by increasing the oligo concentration.

References

[1] Forns, N., De Lorenzo, S., Manosas, M., Hayashi, K., Huguet, J. M., and Ritort, F. (2011) Improving signal/noise resolution in single-molecule experiments using molecular constructs with short handles. Biophysical journal, **100**(7), 1765–1774.

[2] Huguet, J. M., Bizarro, C. V., Forns, N., Smith, S. B., Bustamante, C., and Ritort, F. (2010) Single-molecule derivation of salt dependent base-pair free energies in DNA. Proceedings of the National Academy of Sciences, **107**(35), 15431–15436.
